# Neonatal Mouse Gut Metabolites Influence Cryptosporidium parvum Infection in Intestinal Epithelial Cells

**DOI:** 10.1128/mBio.02582-20

**Published:** 2020-12-15

**Authors:** Kelli L. VanDussen, Lisa J. Funkhouser-Jones, Marianna E. Akey, Deborah A. Schaefer, Kevin Ackman, Michael W. Riggs, Thaddeus S. Stappenbeck, L. David Sibley

**Affiliations:** a Department of Pathology and Immunology, Washington University School of Medicine, Saint Louis, Missouri, USA; b Department of Molecular Microbiology, Washington University School of Medicine, Saint Louis, Missouri, USA; c School of Animal and Comparative Biomedical Sciences, College of Agriculture and Life Sciences, University of Arizona, Tucson, Arizona, USA; Albert Einstein College of Medicine

**Keywords:** 16S rRNA, *Cryptosporidium parvum*, enteric infection, essential nutrient, fatty acid, metabolite, microbiota

## Abstract

The protozoan parasite *Cryptosporidium* sp. is a leading cause of diarrheal disease in those with compromised or underdeveloped immune systems, particularly infants and toddlers in resource-poor localities. As an enteric pathogen, *Cryptosporidium* sp. invades the apical surface of intestinal epithelial cells, where it resides in close proximity to metabolites in the intestinal lumen. However, the effect of gut metabolites on susceptibility to *Cryptosporidium* infection remains largely unstudied. Here, we first identified which gut metabolites are prevalent in neonatal mice when they are most susceptible to Cryptosporidium parvum infection and then tested the isolated effects of these metabolites on C. parvum invasion and growth in intestinal epithelial cells. Our findings demonstrate that medium or long-chain saturated fatty acids inhibit C. parvum growth, perhaps by negatively affecting the streamlined metabolism in C. parvum, which is unable to synthesize fatty acids. Conversely, long-chain unsaturated fatty acids enhanced C. parvum invasion, possibly by modulating membrane fluidity. Hence, gut metabolites, either from diet or produced by the microbiota, influence C. parvum growth *in vitro* and may also contribute to the early susceptibility to cryptosporidiosis seen in young animals.

## Abstract (precis)

*Cryptosporidium* sp. occupies a unique intracellular niche that exposes the parasite to both host cell contents and the intestinal lumen, including metabolites from the diet and produced by the microbiota. Both dietary and microbial products change over the course of early development and could contribute to the changes seen in susceptibility to cryptosporidiosis in humans and mice.

## INTRODUCTION

*Cryptosporidium* sp. has gained notoriety in recent years due to its surprising prevalence as a major enteric diarrheal pathogen in children under 2 years of age in Africa and Southeast Asia (1, 2). The parasite is transmitted by a direct oral-fecal route, often through the ingestion of environmentally resistant oocysts in contaminated water supplies (3). Cryptosporidiosis in humans is primarily caused by two species; Cryptosporidium parvum infects a wide variety of domestic livestock and is transferred to humans as a zoonotic infection, although some subtypes are known to circulate more directly between humans (4, 5). In contrast, Cryptosporidium hominis is almost exclusively transmitted human to human (5, 6). Treatment options for cryptosporidiosis are very limited, as the only FDA-approved drug nitazoxanide is ineffective in immunocompromised patients and not approved for use in children (7).

Numerous studies demonstrate that neonatal animals are highly susceptible to *Cryptosporidium* sp. and that resistance to infection increases with age in mice (8), dairy calves (9), and humans (1, 2). In fact, the Global Enteric Multicenter Study (GEMS) found that, in developing countries, *Cryptosporidium* sp. was the second leading cause of diarrheal episodes in infants (0 to 11 months of age), the third leading cause in toddlers (12 to 23 months of age), and nearly absent in children 2 years and older (1, 2). Why neonatal animals are particularly susceptible to the parasite and what causes them to become resistant as they age are not well understood but could result from changes in the immune system, microbiota, or diet, of which all change dramatically in early life.

Interestingly, the increase in resistance to *Cryptosporidium* infection correlates with time of weaning, when drastic shifts in the diversity and composition of the gut microbiota occur in both neonatal mice and human infants (10, 11). As enteric pathogens, *Cryptosporidium* spp. primarily infect the apical end of small intestinal enterocytes, where they are enveloped by host membranes but remain extracytoplasmic (12, 13). Protrusion of the parasite-containing vacuole into the intestinal luminal space places them near the mucosal layers and associated gut microbiota. In fact, several studies have shown that C. parvum infection alters the microbiota of mice (14, 15), and treatment with a probiotic enhanced C. parvum infection, presumably by altering the microbiota (16). Furthermore, loss of the microbiota in gnotobiotic and antibiotic-treated adult mice results in an increased susceptibility to *Cryptosporidium* infection (17), indicating that a diverse, mature microbiota provides a protective effect against *Cryptosporidium* sp. A recent study comparing different antibiotics revealed that cloxacillin treatment of mice induced changes in the microbiota and altered metabolites with increased susceptibility (18).

Since *Cryptosporidium* sp. spends most of its life cycle inside a host cell, interactions between the parasite and the microbiota are likely mediated through metabolites in the intestinal luminal space. Consistent with this idea, one study showed that high levels of fecal indole, a microbial metabolite, protected human volunteers from infection by C. hominis, as monitored by oocyst shedding (19). While indole appears to inhibit the parasite, it is possible that other gut metabolites may promote *Cryptosporidium* growth. The genomes of C. parvum (20) and C. hominis (21) are highly streamlined, with the loss of many metabolic pathways and the expansion of transporters (22); hence, they must acquire many basic nutrients from their host or surrounding environs. It is possible that metabolites highly enriched in the neonatal gut, either derived from diet or the microbiota, are beneficial to the parasite and that the transition from milk to solid food, which is accompanied by changes in the microbiota, deprives *Cryptosporidium* sp. of an essential nutrient.

In the present study, we undertook a systematic study of the changes in susceptibility of neonatal mice and the correlated change in the collective metabolites found in the lumen of the gut on the growth of C. parvum. Our findings reflect both enhancing and inhibitor activities of metabolites, suggesting that gut metabolites may influence susceptibility to infection during early development.

## RESULTS

### Age-dependent susceptibility to C. parvum in a neonatal mouse model of cryptosporidiosis.

**FIG 1:**
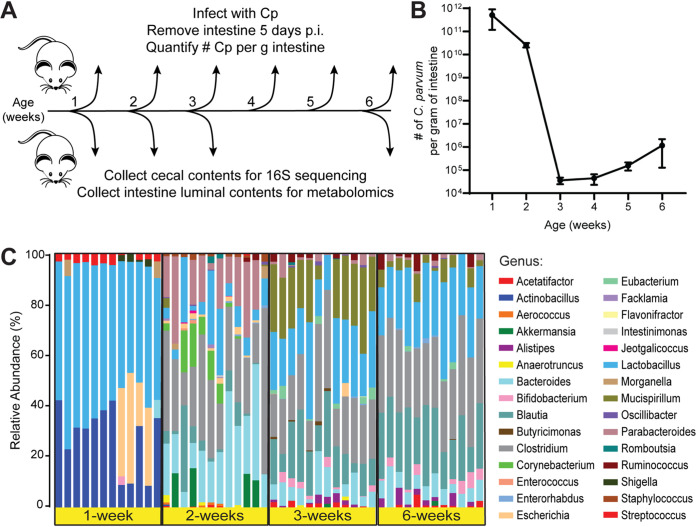
Differences between C. parvum infectivity and cecal microbiota during murine postnatal development. (A) Diagram of the experimental design. Separate cohorts of mice were challenged with 5 × 10^4^ C. parvum oocysts at each week of life (*n* = 5 to 10 mice per week). Five days postinfection (p.i.), intestines were removed, and the number of C. parvum organisms per gram of intestine was quantified by qPCR. In a separate experiment, cecal contents and small intestinal luminal contents were collected from uninfected mice at 1, 2, 3, and 6 weeks of age (*n* = 12 mice per week) for 16S rRNA sequencing and metabolomics, respectively. (B) Line graph depicting the average number of C. parvum organisms per gram intestine of mice infected at the indicated weeks of age (mean ± SD, *n* = 10 mice each for weeks 1 and 2, *n* = 5 mice each for weeks 3 to 6). (C) Taxonomic differences in the cecal microbiota of mice at 1, 2, 3, or 6 weeks of age displayed as a stacked bar graph of the relative abundances of the bacterial genera detected by 16S rRNA sequencing.

To identify gut metabolites that may facilitate *Cryptosporidium* infection, we first determined the critical window of susceptibility to C. parvum in a neonatal mouse model of cryptosporidiosis. Four groups of 10 pups each were reared simultaneously, and a subset of pups was infected each week with 5 × 10^4^ C. parvum oocysts (Fig. 1A, see Fig. S1 in the supplemental material). After 5 days of infection, the number of C. parvum genome equivalents in whole intestines was measured using quantitative PCR (qPCR) and normalized to the initial weight of the intestinal sample (Fig. 1B). Mice infected at 1 week of age had the highest number of C. parvum per gram of intestine, while parasite numbers dropped 10-fold in mice infected at 2 weeks old (Fig. 1B). Mice inoculated at 3 weeks old had the sharpest decline in C. parvum infection, with 5 orders of magnitude less C. parvum per gram of intestine than 1-week-old mice (Fig. 1B). Infection levels remained consistently lower for mice infected at 4, 5, and 6 weeks of age (Fig. 1B), indicating that mice are most susceptible to C. parvum infection within the first 2 weeks of life and experience a drastic reduction in parasite load when infected after this brief window of susceptibility.

10.1128/mBio.02582-20.1 FIG S1 Experimental timeline for testing murine susceptibility to C. parvum and for collecting samples for metabolomics and 16S ribosomal RNA sequencing. In the first set of experiments (black text), mice were infected with 5 × 10^4^ C. parvum oocysts at 1, 2, 3, 4, 5, or 6 weeks of age (*n* = 10 mice each for weeks 1 and 2, *n* = 5 mice each for weeks 3 to 6). Mice were euthanized after 5 days of infection, and all intestinal tissue was removed to quantify the number of C. parvum per gram of intestine using qPCR. In the second set of experiments (purple text), small intestinal luminal flush samples and cecal contents were collected from 12 mice at 1, 2, 3, or 6 weeks of age for metabolomics or 16S ribosomal RNA sequencing, respectively. Download FIG S1, TIF file, 0.8 MB. Copyright © 2020 VanDussen et al. 2020 VanDussen et al. This content is distributed under the terms of the Creative Commons Attribution 4.0 International license.

**FIG 2:**
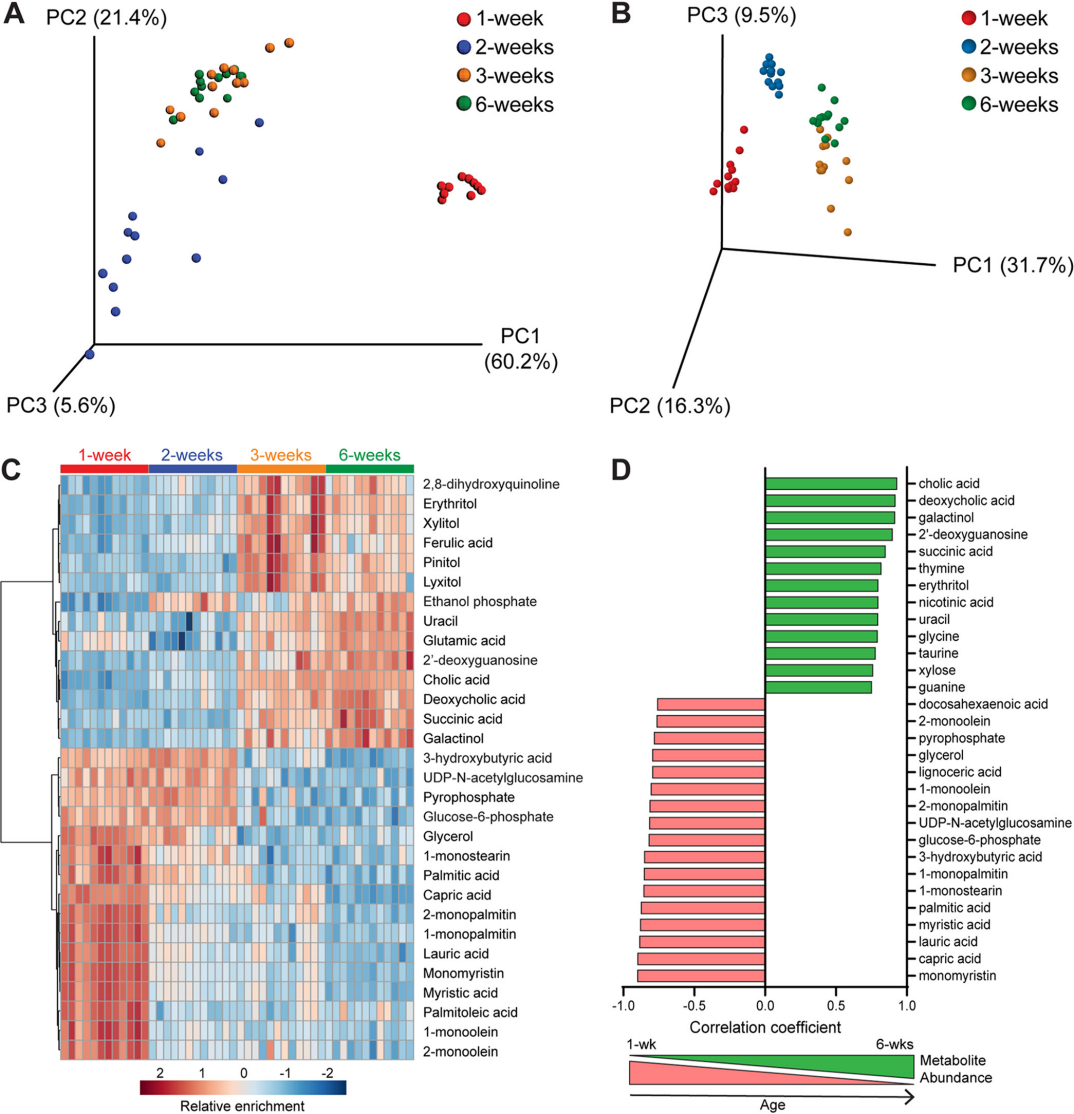
Differences in small intestinal metabolites during murine postnatal development. PCoA plot of weighted Unifrac distances between cecal microbiota samples (A) and PCA plot of small intestinal metabolite differences from the same mice sampled at 1, 2, 3, or 6 weeks of age (B). (C) Hierarchical clustering of the top 30 metabolites that most significantly differed between groups as analyzed by one-way ANOVA, represented as a heat map with red indicating relative enrichment and blue indicating relative de-enrichment for the listed metabolites. (D) Bar graph showing the top 30 metabolites with relative abundances most significantly correlated with age by Pearson’s correlation. Red, negative correlation of metabolite abundance with age; green, positive correlation of metabolite abundance with age.

To verify the course of age-dependent gut microbiome maturation in our model, we collected cecal contents from uninfected mice at time points when they are most susceptible (1 and 2 weeks of age) or relatively resistant (3 and 6 weeks of age) to infection (Fig. 1A, Fig. S1) and performed 16S rRNA sequencing analysis. This analysis revealed drastic changes in the taxonomic composition of microbiota as the mice aged (Fig. 1C), similar to observations of previous studies in neonatal mice (10, 23–25). The microbial communities from 1-week-old mice were the least diverse of all four age groups (see Fig. S2a in the supplemental material) and were dominated by facultative anaerobes from the *Actinobacillus*, *Lactobacillus*, and *Escherichia* genera (Fig. 1C, Fig. S2b and c). By 2 weeks of age, the microbiota had transitioned to mostly strict anaerobes, including *Bacteroides*, *Parabacteroides*, and *Clostridium* (Fig. 1C). In samples from 3-week-old and 6-week-old mice, *Clostridium* remained a significant fraction of the microbiota, while the relative abundances of *Bacteroides* and *Parabacteroides* decreased along with a concurrent rise of the *Blautia* and *Mucispirillum* genera (Fig. 1C, Fig. S2b and c). When all four age groups were analyzed together, a principal-coordinate analysis (PCoA) plot of weighted Unifrac distances showed distinct clusters for 1- and 2-week-old samples, while samples from 3- and 6-week-old mice overlapped (Fig. 2A).

10.1128/mBio.02582-20.2 FIG S2 Cecal microbiota taxonomic differences during murine postnatal development. (A) Cecal microbiome differences in mice aged 1, 2, 3, or 6 weeks assessed by alpha diversity box plots for Chao1 index, Shannon index, or Simpson diversity index. Each box plot represents the median, interquartile range, and maximum and minimum values. Linear discriminant analysis (LDA) effect size (LEfSe) analysis comparing the phylogenetic representations of 1-week versus 6-week age groups as a cladogram (B) or bar graph (C). Inclusion criteria were log effect size of ≥2 and *P* value of <0.05. Download FIG S2, TIF file, 2.9 MB. Copyright © 2020 VanDussen et al. 2020 VanDussen et al. This content is distributed under the terms of the Creative Commons Attribution 4.0 International license.

### Changes in luminal metabolite composition over the first 6 weeks of life.

To identify metabolites that could influence susceptibility to C. parvum infection, we collected small intestine luminal flush samples from the same mice as the microbiome analysis and quantified metabolites present using gas chromatography time of flight mass spectrometry (GC-TOF MS). A principal-component analysis (PCA) plot of metabolite similarities between all samples revealed a similar pattern as that of the microbiome Unifrac analysis; metabolites from 1- and 2-week-old mice formed independent clusters, while those from 3- and 6-week-old mice were interspersed (Fig. 2B). Hierarchical clustering of the 30 metabolites with the lowest false-discovery rate (FDR)-corrected *P* values by one-way analysis of variance (ANOVA) revealed a strong enrichment of fatty acids and their glycerol esters (e.g., myristic acid and monomyristin; palmitic acid and monopalmitin) in 1-week samples only (Fig. 2C). In contrast, several metabolites, such as 3-hydroxybutyric acid, UDP-*N*-acetylglucosamine, and glucose-6-phosphate, were enriched in the first 2 weeks of life but decreased by 3 weeks. As expected given their overlapping PCA clusters (Fig. 2B), 3-week and 6-week samples were mostly enriched for the same metabolites (Fig. 2C) compared to those of earlier time points. However, sugar alcohols, such as erythritol, xylitol, and lyxitol, were generally more abundant at 3 weeks than at 6 weeks, while amino acids (uracil and glutamic acid) and bile acids (cholic and deoxycholic acid) were highest at 6 weeks (Fig. 2C).

A similar, but not identical, pattern emerged when Pearson’s correlation was used to find the top 30 metabolites whose abundances changed linearly over time (i.e., were either positively or negatively correlated with age) (Fig. 2D). The same fatty acids and their glycerol esters that were enriched in 1-week-old samples (Fig. 2C) were negatively correlated with age, with the addition of docosahexaenoic acid and lignoceric acid (Fig. 2D). Similarly, many of the metabolites enriched at the two later time points were positively correlated with age, with the cholic and deoxycholic bile acids having the strongest correlation (Fig. 2D).

### Screening for effects of neonatal metabolites on C. parvum growth *in vitro*.

**FIG 3:**
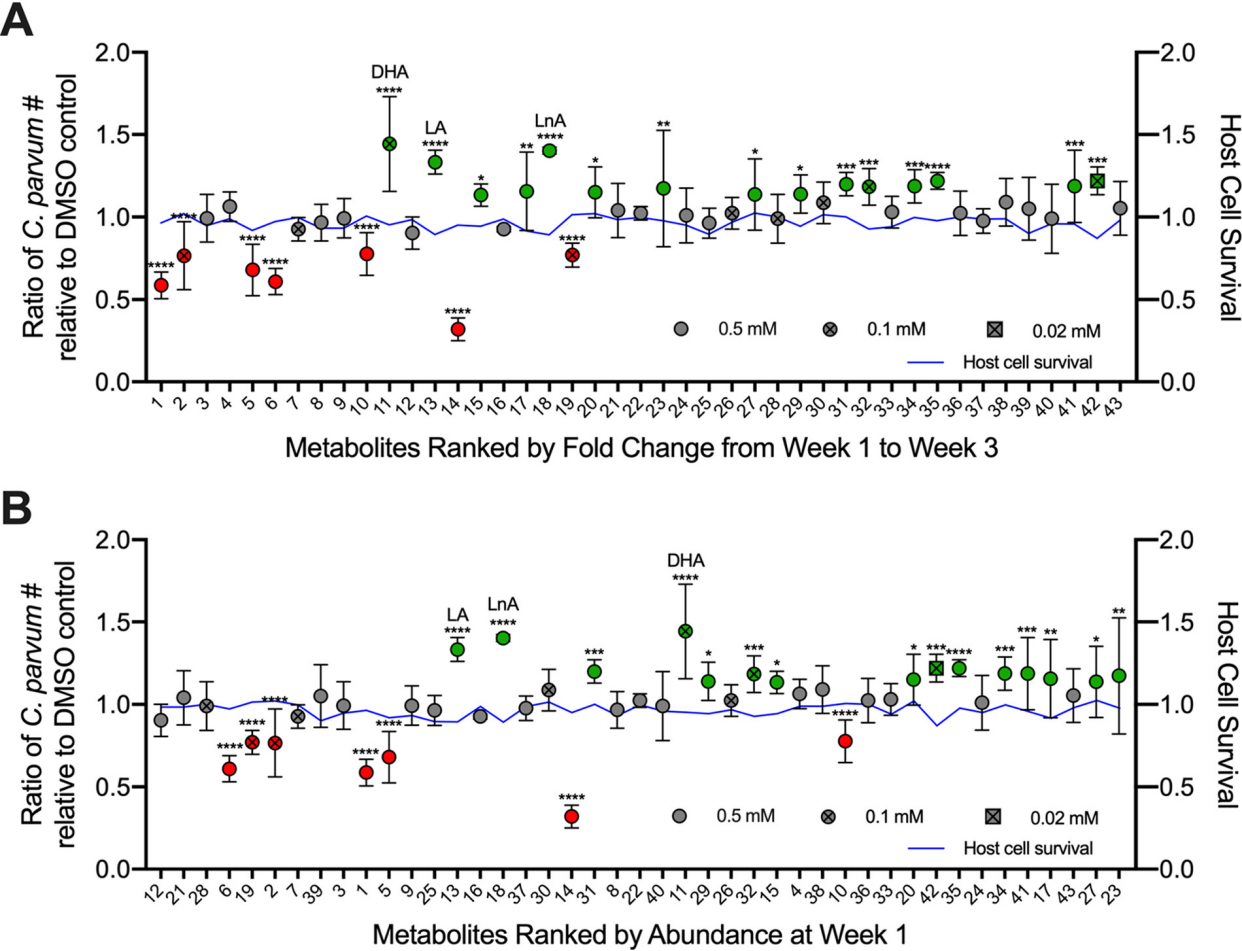
Effects of neonatal metabolites on *Cryptosporidium* growth. Average ratio of C. parvum parasites in treated samples relative to DMSO controls 24 hpi, with metabolites in decreasing order of fold decrease in abundance from mice aged 1 week to mice aged 3 weeks (A) and decreasing order of abundance in mice aged 1 week (B). Metabolites in green were found to significantly enhance growth, and metabolites in red were found to significantly inhibit growth. The three metabolites with the highest fold enhancement of growth are labeled, namely, docosahexaenoic acid (DHA), linoleic acid (LA), and linolenic acid (LnA). Data represent combined mean ± SD of three independent experiments, with 2 to 3 technical replicates per experiment. *, *P ≤ *0.05; **, *P ≤ *0.01; ***, *P ≤ *0.001; ****, *P ≤ *0.0001. The blue line indicates the mean ratio of treated host cells relative to the DMSO control.

To determine if any of the metabolites negatively correlated with age (i.e., highest in 1-week-old samples) were sufficient to enhance C. parvum infection, we screened 43 metabolites for their effect on C. parvum growth in an human ileocecal adenocarcinoma (HCT-8) cell line (see Table S1 in the supplemental material). All metabolites with a negative correlation with age with an FDR-corrected *P* value of <0.05 (Pearson’s test) were included in the screen except for those that proved insoluble or were not readily available for purchase. We also excluded metabolites associated with the microbiota of adult mice that were identified in a previous study comparing germfree to recolonized mice (26). C. parvum growth was quantified using an image-based assay in which C. parvum oocysts were added with a single metabolite to HCT-8 cells plated in a 96-well format. After 24 h of incubation, fixed cells were labeled with Pan-Cp, a polyclonal antibody that recognizes all stages of C. parvum (27) and were stained with Hoechst 33342 to visualize host nuclei. The number of C. parvum and host nuclei in each well were quantified by an automated imaging platform and normalized to dimethyl sulfoxide (DMSO)-treated control wells. All metabolites were first screened at 0.5 mM, a concentration that was chosen based on previous metabolite screening studies (28, 29). However, concentrations were lowered to either 0.1 mM or 0.02 mM for several metabolites that demonstrated significant host toxicity at the higher concentration (Fig. 3, Table 1, and Table S1).

**TABLE 1:** Significant effects of neonatal gut metabolites on C. parvum growth

Rank order of fold change^*a*^	Metabolite name	Concn (mM)	Avg growth ratio (mean ± SD)^*b*^	*P* value
11	Docosahexaenoic acid	0.1	1.44 ± 0.29	<0.0001
18	Linolenic acid	0.5	1.40 ± 0.02	<0.0001
13	Linoleic acid	0.5	1.33 ± 0.07	<0.0001
35	Dihydroxyacetone	0.5	1.22 ± 0.05	<0.0001
42	Behenic acid	0.02	1.22 ± 0.08	0.0003
31	Hexadecane	0.5	1.20 ± 0.07	0.0002
41	Benzylalcohol	0.5	1.19 ± 0.22	0.0006
34	*N*-acetyl-d-mannosamine	0.5	1.19 ± 0.10	0.0006
32	Pentadecanoic acid	0.1	1.18 ± 0.11	0.0009
23	Glucose-6-phosphate	0.5	1.18 ± 0.35	0.0022
17	Diphosphoric acid	0.5	1.16 ± 0.24	0.0089
20	Nicotinamide	0.5	1.15 ± 0.15	0.0150
29	Maltose	0.5	1.14 ± 0.12	0.0310
27	UDP-*N*-acetylglucosamine	0.5	1.14 ± 0.22	0.0390
15	Oxamic acid	0.5	1.13 ± 0.07	0.0488
10	1-Monostearin	0.5	0.78 ± 0.13	<0.0001
2	1-Monopalmitin	0.1	0.77 ± 0.21	<0.0001
19	Palmitic acid	0.1	0.77 ± 0.07	<0.0001
5	Myristic acid	0.5	0.68 ± 0.16	<0.0001
6	Lauric acid	0.5	0.61 ± 0.08	<0.0001
1	Monomyristin	0.5	0.59 ± 0.08	<0.0001
14	Capric acid	0.5	0.32 ± 0.07	<0.0001

a Rank order of metabolites by fold change in abundance from week 1 to week 3 as shown in Table S1.

b Ratio of C. parvum growth relative to the DMSO control averaged across three independent experiments, with two to three technical replicates per experiment, as shown in Fig. 3. Two way ANOVA with Dunnett's test for multiple comparisons.

10.1128/mBio.02582-20.4 TABLE S1 Metabolites screened for their effect on C. parvum growth in HCT-8 cells. Download Table S1, XLSX file, 0.02 MB. Copyright © 2020 VanDussen et al. 2020 VanDussen et al. This content is distributed under the terms of the Creative Commons Attribution 4.0 International license.

Out of the 43 metabolites screened, 7 significantly inhibited C. parvum growth, while 15 significantly enhanced C. parvum infection compared with the DMSO control (Fig. 3). Interestingly, all of the inhibitory metabolites were medium- or long-chain saturated fatty acids and/or their glycerol esters as follows: capric acid (C_10:0_), lauric acid (C_12:0_), myristic acid (C_14:0_) and monomyristin, palmitic acid (C_16:0_) and 1-monopalmitin, and 1-monostearin (C_18:0_) (Table 1). Not all saturated fatty acids were inhibitory, as most had no effect, and two, namely, pentadecanoic acid (C_15:0_) and behenic acid (C_22:0_), modestly enhanced C. parvum growth (Table 1). However, the three most potent enhancers (1.3× to 1.4× growth) were omega-3 or omega-6 polyunsaturated fatty acids, namely, docosahexaenoic acid (DHA; C_22:6_), linolenic acid (LnA; C_18:3_), and linoleic acid (LA; C_18:2_) (Table 1). All the inhibitors and the most effective enhancers (DHA, LA, and LnA) fell within the top 20 metabolites when ranked based on their abundance fold change from week 1 to week 3 (Fig. 3A). When ranked by abundance at week 1, LA and LnA remained in the top 20 metabolites along with all inhibitors except for 1-monostearin (Fig. 3B). Thus, metabolites may have both protective and detrimental effects on susceptibility to C. parvum infection in the neonatal gut.

### Effects of omega-3 and omega-6 polyunsaturated fatty acids on C. parvum growth and invasion.

**FIG 4:**
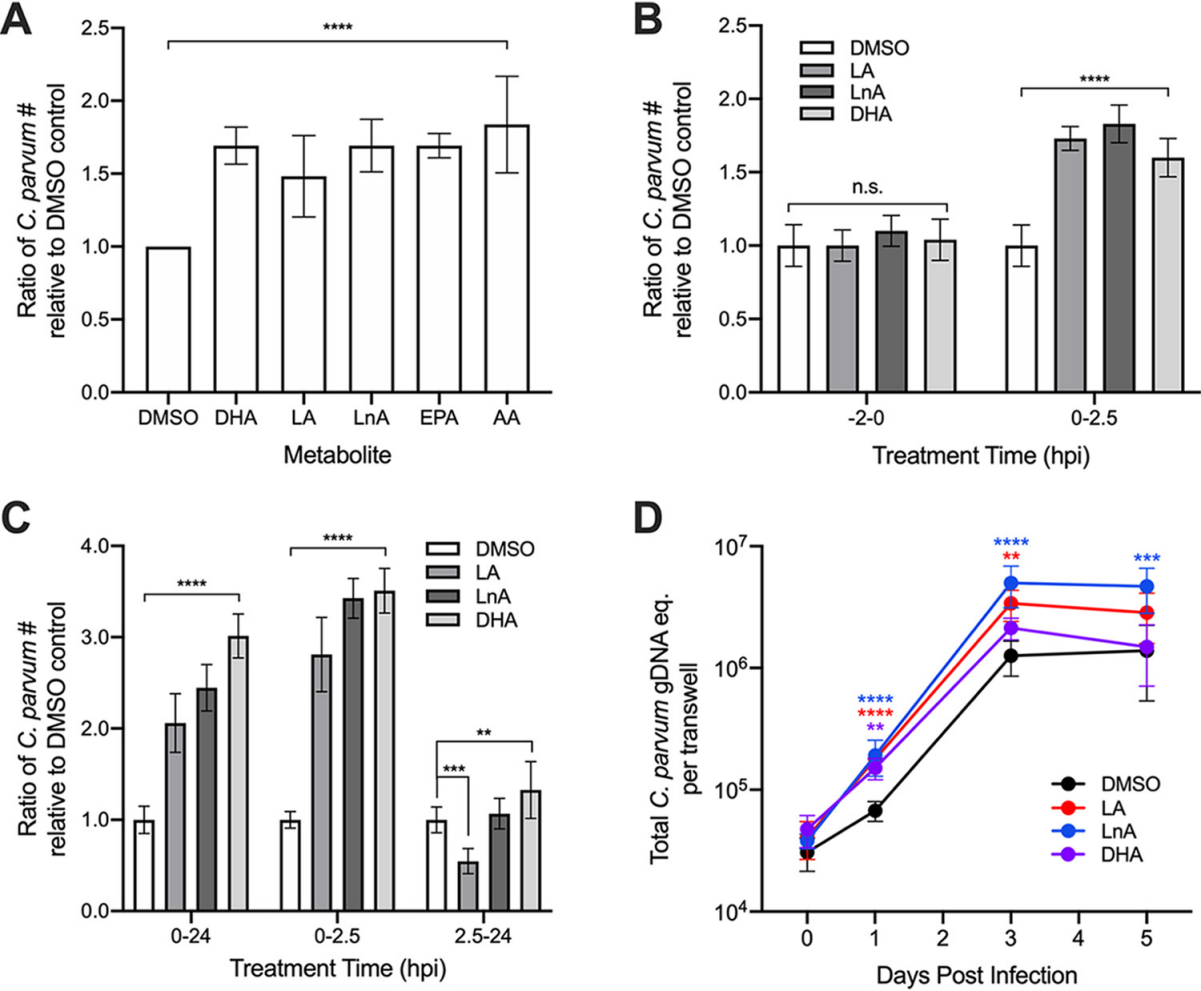
Enhancement of parasite growth and invasion by metabolites and related molecules. (A) Average ratio of C. parvum parasites 24 hpi in treated samples relative to DMSO controls for the metabolites docosahexaenoic acid (DHA), linoleic acid (LA), and linolenic acid (LnA) and the related compounds eicosapentaenoic acid (EPA) and arachidonic acid (AA). (B) Samples were infected with filtered C. parvum sporozoites and washed after a 2.5-h incubation. Average ratio of attached C. parvum parasites relative to DMSO controls compared between samples that were pretreated with metabolites for 2 h and samples with metabolites added immediately after infection. (C) Average ratio of C. parvum parasites relative to DMSO control in samples infected with filtered sporozoites and treated with metabolites during invasion (0 to 2.5 hpi), after invasion (2.5 to 24 hpi), and for the duration of the experiment (0 to 24 hpi). (D) Effects of metabolite treatment on C. parvum growth in air-liquid interface (ALI) culture determined by the average total C. parvum genomic DNA (gDNA) equivalent per transwell on days 0, 1, 3, and 5 postinfection. DHA and AA were tested at a final concentration of 0.1 mM, and all other metabolites were tested at a final concentration of 0.5 mM. All data represent combined mean ± SD of three independent experiments, with 2 to 3 technical replicates per experiment, and were analyzed with a two-way ANOVA followed by a Dunnett’s test for multiple comparisons. **, *P ≤ *0.01; ***, *P ≤ *0.001; ****, *P ≤ *0.0001; n.s., not significant.

As we were most interested in metabolites that may contribute to enhanced susceptibility to C. parvum in neonates, we investigated whether other members of the omega-3 and omega-6 fatty acid families could positively affect C. parvum growth. Indeed, omega-3 eicosapentaenoic acid (EPA; C_20:5_) and omega-6 arachidonic acid (AA; C_20:4_) significantly enhanced C. parvum growth to the same extent as DHA, LA, and LnA in a 24-h growth assay (Fig. 4A), indicating that these two classes of fatty acids have a generally positive effect on C. parvum infection. To investigate whether omega-3 and omega-6 fatty acids affect the invasion efficiency of C. parvum, we infected HCT-8 cells with filtered sporozoites and treated them with either DHA, LA, or LnA during a 2.5-h invasion period. Cells were then extensively washed before fixing, staining, and imaging as described above. All three metabolites significantly increased the number of C. parvum present in the wells compared with the DMSO control, with LA and LnA having a slightly stronger effect than DHA (Fig. 4B). In contrast, parasite numbers did not significantly increase in HCT-8 cells that had been pretreated with DHA, LA, or LnA for 2 h before infection with filtered sporozoites (Fig. 4B). This finding suggests that the fatty acids may be directly facilitating sporozoite adhesion or invasion to host cells, rather than acting through a host signaling pathway to “prime” the cells for invasion.

To determine if the effects of metabolites on parasite growth may be time dependent, samples infected with filtered sporozoites were treated with LA, LnA, or DHA either during invasion (0 to 2.5 hours postinfection [hpi]), after invasion (2.5 to 24 hpi), or for the duration of the experiment (0 to 24 hpi). All samples were washed extensively 2.5 hpi to remove unattached sporozoites, and the culture medium was replaced with or without metabolite solution depending on the respective treatment group. After 24 hpi, all samples were fixed, stained, and imaged as described above. For samples treated with LA, LnA, or DHA, parasite growth was significantly enhanced compared with the DMSO control when cells were treated from either 0 to 2.5 hpi or 0 to 24 hpi (Fig. 4C). However, when treatment began after invasion, treatment with LA significantly inhibited parasite growth, while treatment with LnA had no effect (Fig. 4C). Treatment with DHA from 2.5 to 24 hpi increased parasite numbers relative to the control, but the magnitude of enhanced infection was far lower in samples treated after invasion than in samples where treatment began 0 hpi (Fig. 4C). These results indicate that the enhancement of parasite infection resulting from treatment with LA, LnA, and DHA is dependent on the presence of these metabolites during the first 2.5 hpi. This result implies that the increased parasite numbers observed at later time points may be a direct result of the positive effects of metabolite treatment on sporozoite adhesion or invasion.

Because long-term culture and sexual reproduction of C. parvum is not supported in HCT-8 cells, we tested whether metabolite treatment of parasites grown in a mouse ileal air-liquid interface (ALI) transwell culture (27) would result in enhanced parasite infection. To determine this, transwells containing differentiated mouse intestinal epithelial cells (mIECs) were infected with filtered parasites and treated with LA, LnA, or DHA in both the top and bottom transwell compartments for 3 h. All transwells were then washed to remove unattached sporozoites, and both the top and bottom compartments of each transwell were treated with medium containing either DMSO or metabolite solution for the duration of the experiment. On days 0, 1, 3, and 5 postinfection, DNA samples were collected from transwells, and C. parvum and mIEC genomic DNA quantities were determined using qPCR and standard curve analysis. Treatment with LA or DHA significantly increased parasite numbers relative to the DMSO control at multiple time points, and treatment with LnA significantly enhanced parasite numbers at all time points compared with the control (Fig. 4D). Metabolite treatments did not have adverse effects on epithelial culture, and although they enhanced cell monolayer densities at some time points, this pattern did not correlate with the enhanced growth of C. parvum (see Fig. S3 in the supplemental material). Interestingly, treatment with LnA also increased the fold increase in parasite numbers from day 0 to day 5 by an average of 3-fold relative to the DMSO control, suggesting that the fatty acid may enhance multiple rounds of invasion in long-term culture. As a result, transwells treated continuously with LnA contained significantly greater quantities of C. parvum 5 days postinfection than those treated continuously with DMSO (Fig. 4D).

10.1128/mBio.02582-20.3 FIG S3 Average host cell viability of air-liquid interface (ALI) culture. Effects of metabolite treatment on host cell viability in ALI culture determined by the average total mouse intestinal epithelial cell (mIEC) genomic DNA (gDNA) equivalent per transwell on days 0, 1, 3, and 5 postinfection (mean ± SD; *n* = 8 transwells per timepoint for DMSO; *n* = 9 transwells per timepoint for LA, LnA, and DHA). DHA was tested at a final concentration of 0.1 mM, and all other metabolites were tested at a final concentration of 0.5 mM. Data represent combined mean ± SD of three independent experiments, with 2 to 3 technical replicates per experiment, and were analyzed with a two-way ANOVA followed by a Dunnett’s test for multiple comparisons. *, *P ≤ *0.05; **, *P ≤ *0.01. Download FIG S3, TIF file, 0.1 MB. Copyright © 2020 VanDussen et al. 2020 VanDussen et al. This content is distributed under the terms of the Creative Commons Attribution 4.0 International license.

## DISCUSSION

Neonatal animals, including humans, are highly susceptible to *Cryptosporidium* infection but quickly become resistant to the parasite as they age. In a neonatal mouse model of cryptosporidiosis, we found that susceptibility to the pathogen decreases sharply between 2 and 3 weeks of life, which is concurrent with the cessation of breastfeeding and transition to solid food. This change in diet correlated with drastic shifts in the gut microbiota and luminal metabolites, particularly the reduction of fatty acids typically found in breast milk. Exogenous addition of these fatty acids to *in vitro* cultures revealed that medium- to long-chain saturated fatty acids tend to inhibit C. parvum growth, while omega-3 and omega-6 polyunsaturated fatty acids enhance parasite invasion.

Previous studies in mice demonstrate that the gut microbiota changes dramatically during the first few weeks of life, especially following the dietary transition from breast milk to solid food (10, 24, 25). Specifically, these studies found that neonatal mice were first colonized by facultative anaerobes *Gammaproteobacteria* and *Lactobacillales*, which were progressively replaced by obligate anaerobes *Clostridia* and *Bacteroidia* during and after weaning (10, 24, 25). We observed similar developmental changes in the microbiota of our neonatal mice; in the first week of life, *Lactobacillus* and *Actinobacillus* (a *Gammaproteobacteria*) dominated the community and were replaced by 2 weeks of age with strict anaerobes, including *Clostridium* and *Bacteroides*. *Clostridium* remained a significant fraction of the microbial community in mice postweaning, while *Bacteroides* declined over time. Interestingly, a previous study that colonized germfree mice with cecal contents from neonatal (4 to 12 day) or adult mice (7 weeks) found that *Clostridia* (but not *Bacteroides*) protected mice colonized with adult microbiota against the enteric pathogens Salmonella enterica serovar Typhimurium and Citrobacter rodentium (25). Although the protective mechanism is not fully understood, it was independent of innate and adaptive immune responses but was modulated by metabolites, including succinate (25).

Concurrent with the microbial changes over time, the gut metabolome in our mice also transitioned as they aged; medium- and long-chain fatty acids were abundant in 1- and 2-week-old mice and were gradually replaced with sugar alcohols, amino acids, and bile salts in the 3- and 6-week-old mice. The abundance of fatty acids in preweaned mice reveals the significant contribution of diet to the overall gut metabolome, as fatty acids are important constituents of breast milk (30–33). In contrast, the metabolites enriched by week 6 begin to resemble those found in adult mice (26), and several are metabolic by-products of intestinal bacteria, such as 2,8-hydroxyquinoline (34) and the secondary bile acid deoxycholic acid (35). Hence, the shift in metabolite profiles after weaning is likely due to the absence of milk as a nutrient source along with the production or induction of metabolites by a more mature microbiota. It is important to note that the neonatal gut microbiome and metabolome profiles may vary somewhat between different strains and origins of mice. However, given the predictable shifts in microbiota composition with age (10, 24, 25) and the universal diet of neonatal pups (breast milk), we predict that our major findings of changes in community structure and metabolite abundances over time will also occur in other mouse strains.

Considering the enhanced susceptibility of neonates to C. parvum, we were surprised to find that some medium- to long-chain saturated fatty acids abundant in 1-week-old neonatal mice actually inhibited the growth of C. parvum *in vitro*. C. parvum is thought to lack a system for *de novo* fatty acid synthesis, instead relying on salvage from the host (22). It also lacks β-oxidation and cannot use fatty acids as an energy source (22). However, it contains three isoforms of the enzyme for acyl-coenzyme A (CoA) addition (acyl-CoA synthase [ACS]) (36) needed for activating fatty acids salvaged from the host, a fatty acid synthase (FAS1) that functions as an elongase (37), and a long-chain fatty acid elongase (LCE) (38). Of these enzymes, ACS isoforms prefer saturated substrates of C_12_ to C_18_, and LCE prefers saturated substrates of C_14_ to C_16_ (38). The loading domain of CpFAS1 prefers palmitic acid (C_16:0_), but enzyme activity has been documented with substrates C_12_ to C_24_ as well (39). Given these substrate preferences, it is somewhat surprising that medium-chain fatty acids, such as lauric acid (C_12:0_), myristic acid (C_14:0_), and palmitic acid (C_16:0_), inhibited C. parvum growth *in vitro*. One potential explanation for their inhibitory effects could be if these medium-chain fatty acids inhibit the terminal reductase domain of FAS1, which normally prefers much longer chain substrates (i.e., >C_24_) (37). It is also possible that these fatty acids integrate into parasite membranes and upset the normal balance of lipids, hence compromising cellular functions. These medium-chain fatty acids have also been shown to inhibit bacterial growth *in vitro* (40, 41). Among these inhibitory compounds, capric acid, the strongest growth inhibitor in the 24 h growth assay, has also been shown to inhibit Candida albicans growth and biofilm formation by altering gene expression (42), suggesting it has broad cross-phylum activity. Additional studies are necessary to discern the role that these saturated fatty acid metabolites may have in modulating C. parvum infection.

In contrast to the inhibitory saturated fatty acids, long-chain, omega-3, or omega-6 polyunsaturated fatty acids, linoleic acid (LA; C_18:2_), linolenic acid (LnA; C_18:3_), and docosahexaenoic acid (DHA; C_22:6_) were all significant enhancers of parasite growth *in vitro*. Interestingly, enhancement was also dependent on the timing of exposure; although pretreatment of host cells had no effect on subsequent infection, exposure during the first 2.5 h of infection was critical to the enhancing effect. This timing implies that fatty acid metabolism may not be responsible for the observed increase in parasite infection since these polyunsaturated fatty acids had minimal effects when added after invasion. However, these results suggest that polyunsaturated fatty acids may directly enhance invasion and/or formation of the parasitophorous vacuole that encases the parasite (12, 13). Since invasion and vacuole membrane formation require reorganization of host and parasite membranes in a rapid process of envelopment (43–45), the enhancing effects of these long-chain, unsaturated fatty acids may reflect the important properties they have on membrane composition, fluidity, and signaling (46, 47).

Although our studies were performed *in vitro*, they could have important implications for *Cryptosporidium* infections *in vivo*. The human microbiota undergoes similar predictable transitions as mice, from facultative aerobic bacteria such as *Enterobacteriaceae* at birth to organisms that specialize on a milk-based diet, such as *Lactobacillus*, and then finally to a more mature, “adult-like” microbiota by 2 to 3 years of age (48–50). Interestingly, the microbiotas of children breast-feeding at 12 months old are still dominated by *Bifidobacterium* and *Lactobacillus*, while the microbiotas of children that have stopped breast-feeding by this age are enriched in species prevalent in adults such as *Clostridia* (48). This information suggests that the main driver of microbiota maturation is the cessation of breast-feeding and highlights the importance of breast milk in shaping the overall gut microbiota and metabolome. In our mice, polyunsaturated fatty acids in the gut lumen decreased significantly following weaning. Although fatty acid profiles in breast milk vary between species, all mammals produce essential fatty acids LA and LnA in their breast milk, as well as significant amounts of long-chain unsaturated fatty acids, such as AA and DHA (33). Our finding that LA, LnA, AA, and DHA all enhance sporozoite invasion suggests the possibility that human infants who are nursing may be more susceptible to *Cryptosporidium* infection due to higher levels of these metabolites in their guts in comparison to older, weaned children. Unfortunately, due to difficulty in sampling and variability in intestinal contents over a 24-hour period, we cannot say whether the concentration of polyunsaturated fatty acids needed to enhance C. parvum growth in our *in vitro* assays would be physiologically relevant *in vivo*. However, future studies in mice could test whether exogenous administration of these fatty acids, either directly through gavage or indirectly by changing the maternal diet, would affect infection levels.

Although our study suggests a role for gut metabolites in modulating C. parvum infection, there are likely other factors that contribute to the increased susceptibility of neonatal mice and humans to *Cryptosporidium* sp. In particular, maturation of the immune system plays an important role in the decrease of susceptibility to infection during early life. In regard to *Cryptosporidium* sp. specifically, CD103^+^ CD11c^+^ dendritic cells (DCs) are found at low levels in neonatal mice and increase with maturation and during infection. Selective depletion of CD103^+^ dendritic cells in Batf3 knockout mice (51), or increase in their number by delivery of the Flt-3 ligand (52), suggest that changes in these innate immune cells may underlie changes in susceptibility to C. parvum during maturation. Interestingly, administration of poly(I·C) to neonatal mice stimulated immune responses, including expanded DC functions, that required the presence of gut flora (53), indicating that the microbiota and immune function are tightly linked during early development. Thus, the findings of our study provide a framework for future studies to tease apart the potential effects of diet, the microbiota, and the immune system on the susceptibility of infants to cryptosporidiosis and possibly other enteric infections.

## MATERIALS AND METHODS

### Neonatal mouse model of C. parvum infection.

For infections of neonatal mice performed at the University of Arizona, C. parvum (Iowa strain) (54) oocysts were maintained by repeated passage in newborn *Cryptosporidium*-free Holstein bull calves (55) and purified from fecal material by sucrose density gradient centrifugation, as previously described (56).

To assess C. parvum infection levels with age *in vivo*, groups of 5 to 10 8-day-old specific-pathogen-free ICR mice (Envigo) were used. All mice used in the present study were maintained in biosafety level 2 (BSL2) biocontainment at the University of Arizona in accordance with the PHS Guide for the Care and Use of Laboratory Animals and IACUC approval.

Neonatal mice were randomly assigned to litters as detailed in Fig. S1. At 1-week intervals after birth, mice were gavaged with 5 × 10^4^ C. parvum (Iowa strain) oocysts (*n* = 10 mice each for 1 and 2 weeks of age, *n* = 5 mice each for 3 to 6 weeks of age). At 5 days postinfection (92 to 94 h postinfection), the entire intestine was extracted from each mouse, weighed, and then homogenized using ceramic beads in the Bead Ruptor4 system (Omni International, Kennesaw, GA). DNA was extracted using the QIAamp fast DNA stool minikit (Qiagen, Gaithersburg, MD) with the following modifications: after the addition of InhibitEx buffer, the samples were incubated at 95°C (5 min), followed by 5 freeze-thaw cycles using liquid nitrogen and a 37°C water bath. Total DNA in the samples was quantified by a Nanodrop instrument (Thermo Scientific, Waltham, MA).

Quantitative PCR (qPCR) for the C. parvum 18S rRNA was performed using the following primers: ChvF18S (5′-CAATAGCGTATATTAAAGTTGTTGCAGTT-3′) and ChvR18S (5′-CTGCTTTAAGCACTCTAATTTTCTCAAA-3′) (57) For qPCR, each 25-μl reaction contained a final concentration of 100 nM for both forward and reverse primers (Invitrogen, Grand Island, NY) and 12.5 μl SYBR green fast mix (Quantabio, Gaithersburg, MD). Genomic DNA (2 μl) was added, and the qPCR was performed in an ABI StepOnePlus real-time PCR system (Applied Biosystems, Grand Island, NY) with the following cycling conditions: 10-min incubation at 94°C, followed by 45 cycles at 94°C for 10 sec, 54°C for 30 sec, and 72°C for 10 sec. Each sample was run in triplicate. A control with no template was run concurrently and was consistently negative. The number of C. parvum genomic equivalents was calculated for each sample based on a standard curve using DNA from known quantities of C. parvum oocysts and divided by the original weight of the intestinal sample to obtain the number of C. parvum organisms per gram intestine.

### Sample collection for 16S sequencing and metabolomics.

Six pregnant ICR dams with litter sizes of 10 pups each were obtained from the same source (Envigo) as that used for the neonatal infection experiment. Dams and the resulting pups were maintained in a specific-pathogen-free barrier facility at Washington University School of Medicine with a strict 12-h light cycle and *ad libitum* access to food and water. Mice were housed in complete autoclaved cage assemblies containing the same chow (Envigo NIH-31 Irradiated Modified Open Formula Mouse/Rat Diet 7913) and bedding (Envigo Teklad 7097 1/4” Corncob bedding) used in the neonatal infection experiment. To minimize experimental variation that could potentially arise from single cages or dams, 2 pups were randomly selected from each litter per time point (total of *n* = 12 per time point) at 1 week, 2 weeks, 3 weeks, 4 weeks, and 6 weeks of age. Weaning was performed as usual at 3 weeks of age, with pups of the same sex housed only with littermates in fresh autoclaved cage assemblies. All procedures were approved by the Institutional Animal Care and Use Committee at Washington University School of Medicine. For the collection of small intestinal luminal flushings for metabolomics, pups were euthanized and then the entire length of small intestine was dissected intact and flushed with 500 μl of sterile phosphate-buffered saline (PBS) using a 1-ml syringe tipped with a blunt needle; small intestinal luminal contents from the flush were collected directly into a tared cryotube, weighed, and snap-frozen in liquid nitrogen. For collection of cecal contents for 16S rRNA sequencing, the intact cecum was dissected and placed into a tared cryotube (pups aged 1, 2, or 3 weeks), or cecal contents were collected using a sterilized spatula and placed into a tared cryotube (pups aged 4 or 6 weeks); the material was weighed and then snap-frozen in liquid nitrogen.

### 16S sequencing and analysis.

DNA from cecal contents was isolated using the QIAamp DNA stool minikit (Qiagen). The Washington University Genome Technology Access Center performed PCR amplification of all nine 16S variable regions with the Fluidigm access array system, by indexing, pooling, and sequencing with an Illumina MiSeq sequencer, as previously described (58). Sequencing data analysis either used the V1 to V9 regions and the MVRSION pipeline (59) or the V4 region and QIIME pipeline version 1.9.0 (60), as previously described (58). The operational taxonomic unit (OTU) table resulting from QIIME analysis was used as the input for linear discriminant analysis (LDA) effect size (LEfSe) (61) (http://huttenhower.sph.harvard.edu/lefse/) to identify statistically significant, differentially abundant taxa between the 1-week-old and 6-week-old mice.

### Metabolite profiling and analysis.

Untargeted metabolomics of the small intestinal luminal flushing samples by GC-TOF mass spectrometry was performed by the West Coast Metabolomics Center using the primary metabolism platform and a Leco Pegasus IV mass spectrometer. Of the 759 metabolites identified, 213 were annotated and used for further analysis. Data were normalized across samples by averaged week 1 values, before being log_2_-transformed and autoscaled. Data normalization and downstream univariate, multivariate, and clustering analyses were performed with MetaboAnalyst 3.0 (https://www.metaboanalyst.ca) (62).

### HCT-8 cell culture and infection.

For *in vitro* infection studies in human cell lines, C. parvum (AUCP-1 strain) oocysts were obtained from the Witola lab at the University of Illinois at Urbana-Champaign, where they were maintained by repeated passage in male Holstein calves and purified from fecal material as previously described (63). Animal procedures were approved by the Institutional Animal Studies Committee at the University of Illinois at Urbana-Champaign. Purified oocysts were stored at 4°C in PBS plus 50 mM Tris and 10 mM EDTA (pH 7.2) for up to 6 months before use.

Human ileocecal adenocarcinoma cells (HCT-8 cells; ATCC CCL-244) were maintained in RPMI 1640 medium (Gibco; ATCC modification) supplemented with 10% fetal bovine serum. Cells were confirmed to be mycoplasma free with the e-Myco plus *Mycoplasma* PCR detection kit (Boca Scientific).

### C. parvum growth assay for initial metabolite screen.

Metabolites were chosen for the screen based on a negative Pearson’s coefficient and an FDR *P* value of ≤0.05. Metabolites that were insoluble or not readily available for purchase were excluded. We also excluded metabolites that had previously been shown to be present in the gut metabolome of germfree mice and, thus, not likely produced or induced by the microbiota (26). In total, we tested 43 metabolites for their effects on C. parvum growth (Table S1).

All metabolites (Sigma-Aldrich) were reconstituted as 100 mM stock solutions in DMSO with the following exceptions: glucose-6-phosphate was dissolved in filtered PBS, phosphoethanolamine and glycerol-alpha-phosphate were dissolved in filtered dH_2_O, and cholesterol and arachidic acid were dissolved in filtered ethanol. HCT-8 cells were plated at 2 × 10^5^ cells per well in 96-well optically clear-bottomed plates (Greiner Bio-One) and infected with 1.2 × 10^4^ to 5 × 10^4^ C. parvum oocysts (AUCP-1 strain) per well after 24 h of cell growth. Metabolites were diluted in culture medium and immediately added to the wells following the addition of oocysts for a final metabolite concentration of 0.02 mM to 0.5 mM (depending on the metabolite) (Table S1) and 0.5% DMSO (three technical replicate wells per metabolite). Infected control wells containing only 0.5% DMSO media were included on each plate. At 24 h after infection, wells were fixed in 4% formaldehyde for 10 min, washed twice with PBS, and then permeabilized and blocked for 20 min in blocking buffer composed of 0.1% Triton X-100 and 1% bovine serum albumin (BSA) in PBS. C. parvum bacteria were labeled with polyclonal rabbit anti-Cp antibody (27) diluted 1:2,000 in blocking buffer, followed by goat anti-rabbit Alexa Fluor 488 (1:1,000, Thermo Fisher Scientific). Host nuclei were stained with Hoechst 33342 (5 μg/ml; Thermo Fisher Scientific).

Plates were imaged with a 10× objective on a BioTek Cytation 3 cell imager (9 images per well in a 3 by 3 grid). Gen5 software version 5.0.2 was used to quantify the total number of parasites (puncta in the green fluorescent protein [GFP] channel) and host cells (nuclei in the 4′,6-diamidino-2-phenylindole [DAPI] channel) in images from each well. Relative parasite growth and host cell viability for each metabolite were calculated as a ratio of the mean number of C. parvum parasites or host cells, respectively, in the treated versus DMSO control groups averaged across three independent experiments, with three technical replicates per experiment. Statistical analyses were performed in GraphPad Prism 8 using a two-way ANOVA followed by a Dunnett’s test for multiple comparisons, in which each metabolite was compared to the DMSO control.

### C. parvum invasion assay.

HCT-8 cells were plated at 2 × 10^5^ cells per well in 96-well optically clear-bottomed plates (Greiner Bio-One) and cultured for 24 h as described above. To determine the effect of metabolite treatment on host cells before the addition of parasites, metabolite solutions diluted in culture media were added to half of the plate for a final concentration of 0.1 mM to 0.5 mM (depending on the metabolite) (Table S1) and 0.5% DMSO for 2 h and then washed 3× with PBS. Bleached C. parvum oocysts (AUCP-1 strain) were excysted for 1 h at 37°C in a 0.75% sodium taurocholate solution and passed through a 1-μm filter to remove unexcysted oocysts. All wells were infected with approximately 2 × 10^5^ excysted sporozoites. Metabolite solutions diluted in culture media were then added to the second half of the plate for a final concentration of 0.1 mM to 0.5 mM and 0.5% DMSO. Control wells containing only 0.5% DMSO culture media were included for each half of the plate at each time point. After 2.5 h of infection, wells were fixed and stained with polyclonal rabbit anti-Cp antibody (1:5,000), goat anti-rabbit Alexa Fluor 488 (1:1,000; Thermo Fisher Scientific), and Hoescht 33342 (5 μg/ml; Thermo Fisher Scientific) as detailed above.

Parasites and host cells were imaged and quantified using the same protocol as the C. parvum growth assay. Relative parasite growth and host cell viability for each metabolite were calculated as a ratio of the mean number of C. parvum parasites or host cells, respectively, in the treated versus DMSO control groups averaged across three independent experiments, with three technical replicates per experiment. Statistical analyses were performed in GraphPad Prism 8 using a two-way ANOVA followed by a Dunnett’s test for multiple comparisons, in which each metabolite was compared to the DMSO control within each treatment group.

### C. parvum invasion wash-out assay.

HCT-8 cells were plated 2 × 10^5^ cells per well in a clear-bottomed 96-well plate. After 24 h of cell growth, cells were infected with 1 × 10^5^ filtered, excysted sporozoites per well. Immediately after infection, metabolite solutions (0.5 mM except for DHA that was 0.1 mM) or DMSO control were added to wells. After 2.5 h of incubation, all wells were washed 2× with PBS, and metabolite solutions or DMSO control were added to wells as appropriate for each group. At 24 hpi, all wells were fixed and stained as described for the C. parvum invasion assay above.

Parasites and host cells were imaged and quantified as detailed in the C. parvum growth assay. Relative parasite growth and host cell viability for each metabolite were calculated as a ratio of the mean number of C. parvum parasites or host cells, respectively, in the treated versus DMSO control groups averaged across three independent experiments, with three technical replicates per experiment. Statistical analyses were performed in GraphPad Prism 8 using a two-way ANOVA followed by a Dunnett’s test for multiple comparisons, in which each metabolite was compared to the DMSO control within each treatment group.

### Quantification of C. parvum in metabolite-treated air-liquid interface transwells.

Mouse intestinal epithelial cell (mIEC) monolayers were cultured on transwells with an air-liquid interface (ALI) as previously described (27, 64). Briefly, irradiated 3T3 mouse fibroblast cells (CRL-1658 ATCC) were plated on transwells (polyester membrane, 0.4-μm pore; Corning Costar) coated with 10% Matrigel (Corning) and cultured at 37°C for approximately 24 h in Dulbecco’s modified Eagle’s medium (DMEM; high glucose; D6429; Sigma) with 10% fetal bovine serum (Sigma) and 1× penicillin/streptomycin (Sigma). Primary mouse ileal stem cells were harvested from 3-day-old spheroid cultures in Matrigel, dissociated with trypsin as previously described (65), and plated onto irradiated i3T3 monolayers at 5 × 10^4^ mIECs per transwell. mIEC monolayers were cultured with 50% L-WRN-conditioned medium (66) and 10 μM Y-27632 ROCK inhibitor (Torcis Bioscience) in both the top and bottom compartments of the transwell for 7 days, after which the medium was removed from the top compartment to create the air-liquid interface. Three days after removal of the top medium, each transwell was infected with 2 × 10^5^ filtered, excysted sporozoites, and DMSO control or metabolite solutions (0.5 mM except for DHA that was 0.1 mM) were added to both the top (50 μl) and bottom (400 μl) compartments of the transwell. After 3 h of incubation, the top medium was removed, and each transwell was washed with PBS. Each transwell was then treated continuously with either DMSO control or metabolite solution in both the top and bottom chamber for the duration of the experiment.

DNA from transwells was collected and extracted using the QIAmp DNA minikit (Qiagen). qPCR was performed using the QuantStudio 3 system with cycling conditions of a 10-min incubation at 95°C and then 40 cycles at 95°C for 15 s and 60°C for 1 min, followed by a continuous melt curve analysis to identify samples with evidence of nonspecific amplification. Each reaction contained 2 μl purified transwell DNA (diluted 1:5) as a template, 10 μl SYBR green QuickStart *Taq* ReadyMix (Sigma), and 1.6 μl of 5 μM primer solution targeting C. parvum GAPDH (forward, 5′-CGGATGGCCATACCTGTGAG-3′; reverse, 5′-GAAGATGCGCTGGGAACAAC-3′) (27) or mouse GAPDH (forward, 5′-GCCATGAGTGGACCCTTCTT-3′; reverse, 5′-GAAAACACGGGGGCAATGAG-3′) (27). Each transwell sample was run with technical duplicates, and negative (water) controls were included in each plate.

C. parvum and mIEC genomic DNA (gDNA) quantities per transwell were determined via the QuantStudio Design & Analysis New (DA2) software using standard curves for C. parvum and mouse gDNA, respectively. Total C. parvum or mIEC gDNA per transwell was calculated as an average of gDNA quantities per transwell across three independent experiments with two to three technical replicates per experiment. Statistical analyses were performed in GraphPad Prism 8 using a two-way ANOVA followed by a Dunnett’s test for multiple comparisons, in which each metabolite was compared with the DMSO control within each time point.

### Data availability.

The 16S rRNA sequencing reads are available in the ArrayExpress database (http://www.ebi.ac.uk/arrayexpress) under accession number E-MTAB-9100. All remaining data discussed in this report are found in the main figures or the supplemental materials.
