# Supplementary figures and images for: Neonatal Mouse Gut Metabolites Influence Cryptosporidium parvum Infection in Intestinal Epithelial Cells
Source: mBio. 2020 Dec 15;11(6):e02582-20. doi: 10.1128/mBio.02582-20 (PMC7773987; doi:10.1128/mBio.02582-20)

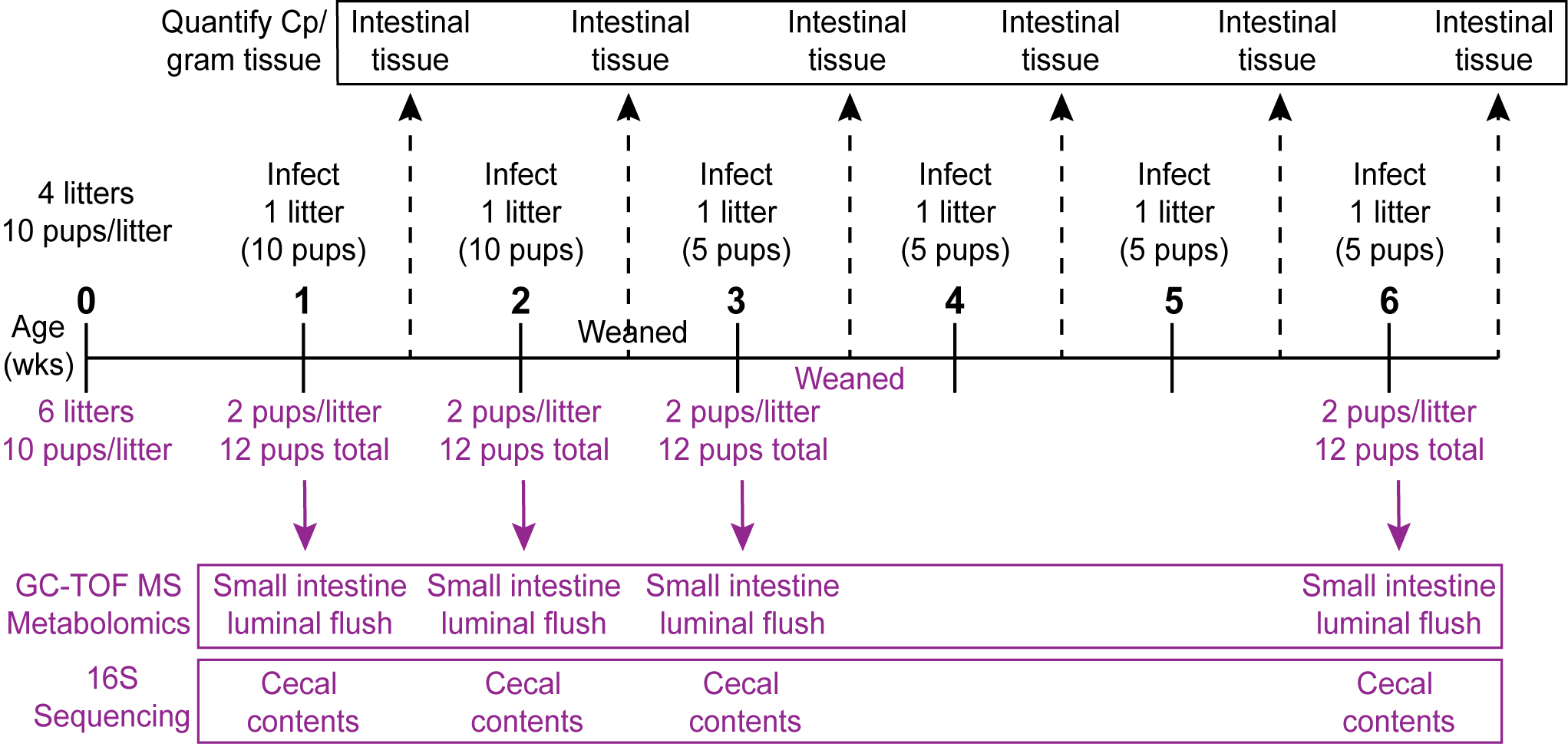

Supplement: FIG S1 [file mBio.02582-20-sf001.tif]

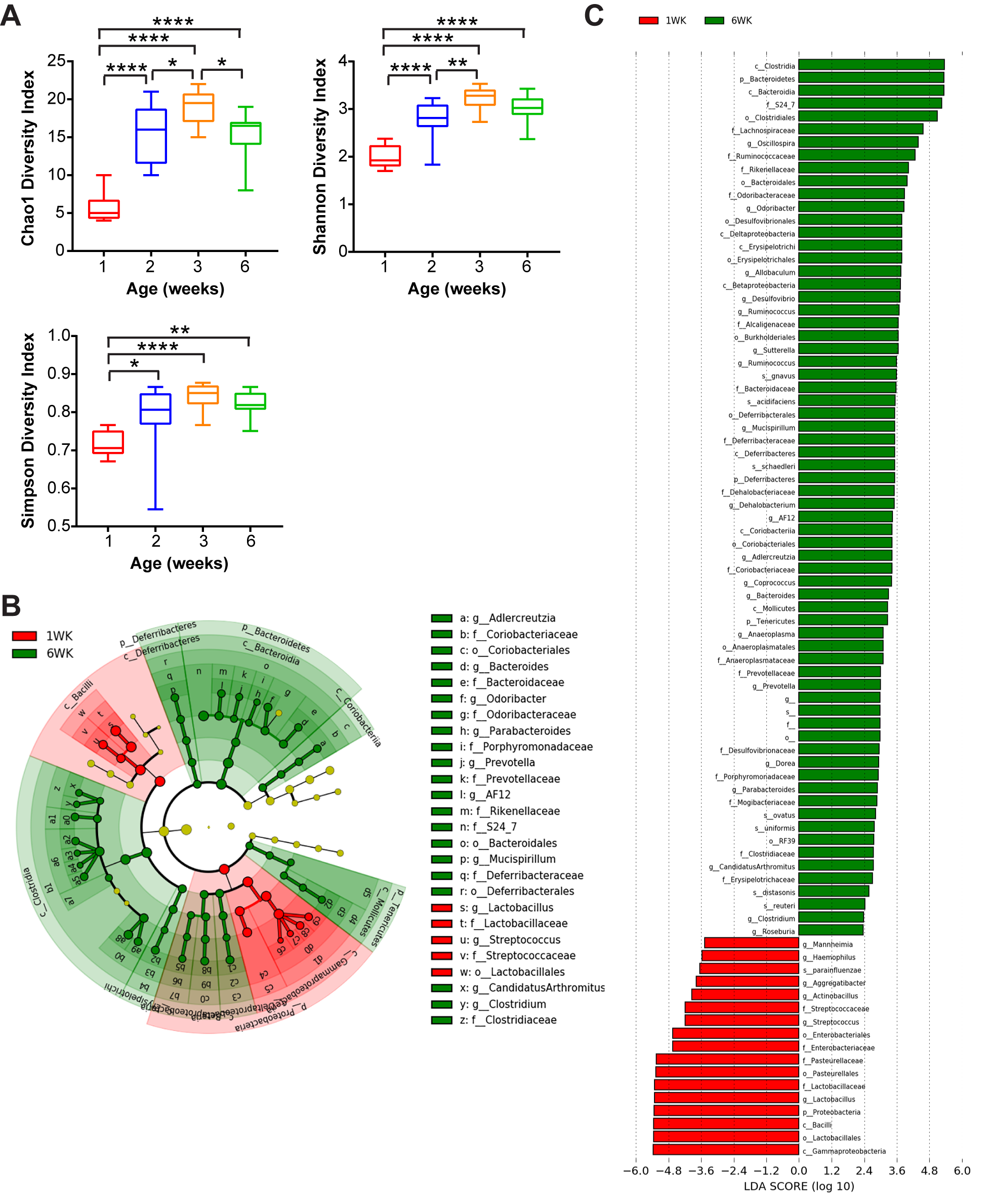

Supplement: FIG S2 [file mBio.02582-20-sf002.tif]

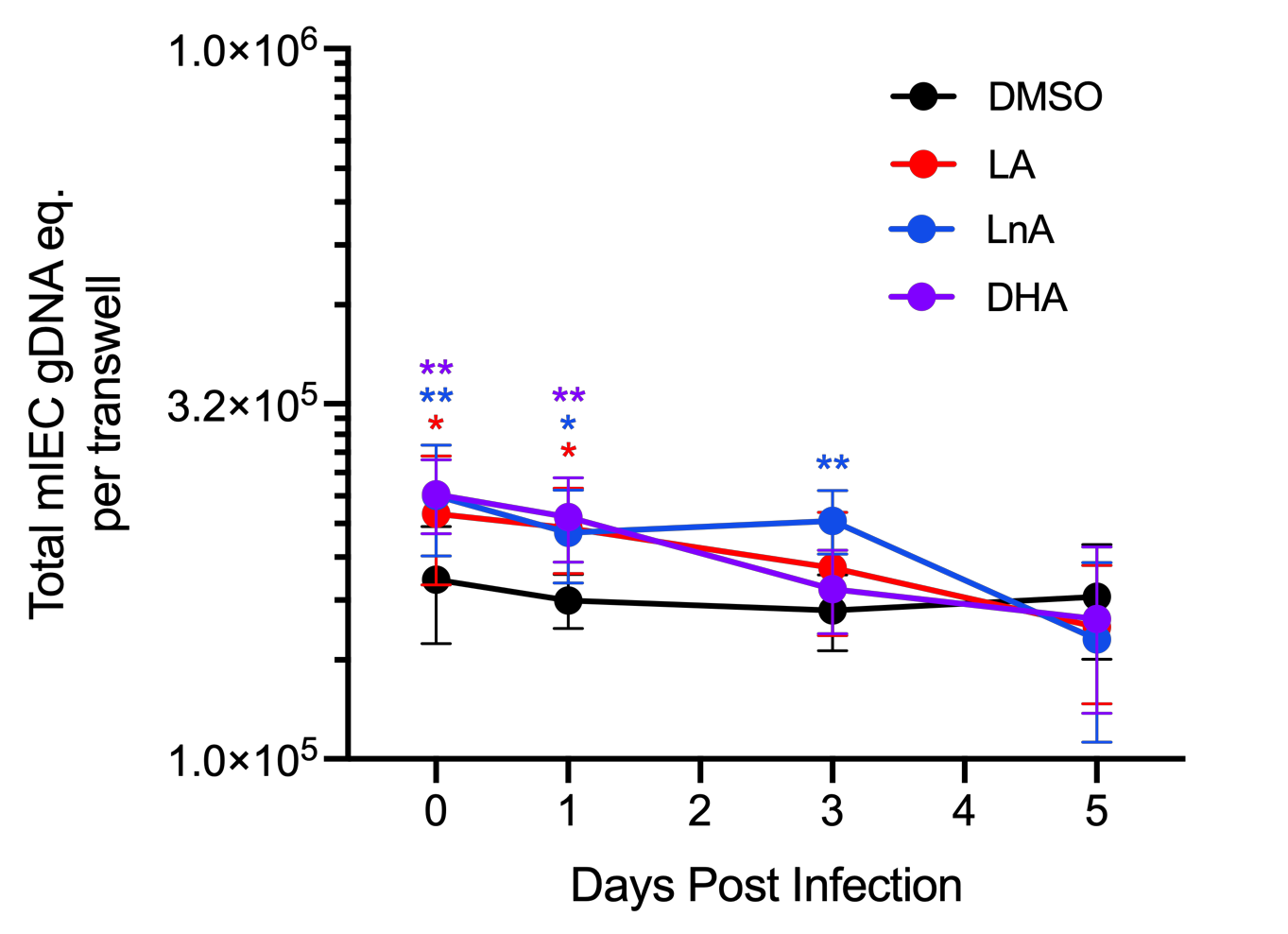

Supplement: FIG S3 [file mBio.02582-20-sf003.tif]
